# Study on the mechanism of visual aging in cats’ primary visual cortex based on BDNF-TrkB signal pathway

**DOI:** 10.1038/s41598-022-14918-z

**Published:** 2022-06-22

**Authors:** Chuanwang Tong, Senyang Cao

**Affiliations:** 1 College of Food and Bioengineering, Wuhu Institute of Technology, Wuhu, 241003 People’s Republic of China; 2 Center of Reproductive Medicine, Huai’an Maternal and Child Health Care Hospital, Huai’an, 223002 People’s Republic of China

**Keywords:** Neurological disorders, Neural ageing

## Abstract

To explore the expression of brain-derived neurotrophic factor (BDNF) and specific receptor tyrosine kinase receptor B (TrkB) in the primary visual cortex of young and old cats, especially to reveal the age-related differences in the mediating mechanism of BDNF-TrkB signaling pathway in cats’ visual cortex and their possible effects on synaptic plasticity, Nissl staining was used to display neurons in each layer of cats’ primary visual cortex, and immunohistochemical ABC method was used to label BDNF and TrkB immunopositive cells in each layer of cats’ primary visual cortex. The BDNF and TrkB receptor immunoreactive neurons and non-neurons were observed and photographed. Their density and immunoreactive intensity were measured. Results showed that BDNF and TrkB were widely expressed in all layers of visual cortex in young and old cats. Compared with the young group, the density and intensity of BDNF and TrkB positive cells in each layer of primary visual cortex in the old group decreased significantly (*P* < 0.01). The findings indicate that the expression levels of BDNF and TrkB in the primary visual cortex of cats decrease with age, suggesting that the change of BDNF-TrkB signal pathway caused by the weakening of brain-derived neurotrophic factor activity may be one of the important reasons for the decline of visual function.

## Introduction

Visual function is an important way for higher mammals to obtain external information. With the growth of age, visual function will also have a nonpathological decline^1^. The research on the neural mechanism of visual aging has always been one of the focuses of brain mechanism research. Brain-derived neurotrophic factor (BDNF) is expressed in the central nervous system and has the most content in mammalian brain. It is mainly distributed in key brain regions such as hippocampus and cortex, of which CA3 region of hippocampus is the most abundant. Tyrosine kinase receptor B (TrkB) is a membrane-bound receptor. BDNF can interact with high affinity receptor TrkB and play an important role in the nervous system. Studies have shown that BDNF and its downstream pathways play a variety of roles in the central nervous system and are related to the development of neurodegenerative diseases such as Alzheimer's disease(AD), Parkinson's syndrome(PD) and Huntington's disease(HD)^2–5^. The mechanism of BDNF and its specific receptor TrkB signaling pathway on brain functional activities has been noticed. It is found that BDNF-TrkB signaling pathway is an important regulatory factor involved in the mechanisms of neuronal development, survival, injury repair and synaptic plasticity of the nervous system^6–8^. Studies on the expression of BDNF and its receptors in mammalian visual system have been reported, such as the relationship between BDNF and visual deprivation patterns in the process of visual development plasticity; The key role of BDNF in synaptic reorganization of visual cortex; The regulatory effect of BDNF and its receptor TrkB on GABA (gama-aminnobutyric acid) ergic neurons during the critical period of visual development^9–11^. However, the expression and possible mechanism of BDNF and TrkB in mammalian primary visual cortex related to visual aging are rarely reported.

In this study, the primary visual cortex of cats was used as the research object because the evolution of cat's visual system is relatively advanced and has better similarity in organizational structure and system function compared with human beings. Therefore, cat is also a more common model animal for visual system related research. Nissl staining methods were used to show layering structure in the primary visual cortex. Immunohistochemical methods were used to compare the expression of BDNF and TrkB in the primary visual cortex of young and old cats, the experimental data results are observed and quantitatively compared to reveal the influencing factors and possible mechanism of visual function decline at the level of visual cortex.

## Materials and methods

### Ethics statement

The ethics of this study was approved by Wuhu Institute of Technology Academic Committee. All experimental procedures were strictly performed in accordance with the ethical requirements of Wuhu Institute of Technology Academic Committee for the use of laboratory animals.

### Animal subjects and experimental reagents

8 Cats (Domestic cat, *Felis catus*) were selected as experimental animals, with an individual weight of 2–3.5 kg (healthy, male). The experimental animals were divided into two groups according to their age: young group and old group. 4 cats (1–3 years old) were selected to form the young group and 4 cats (10–13 years old) were selected to form the old group. The experimental cats were purchased from the animal farm in Jiangning District, Nanjing, China (license No.SX1207). The cats were raised for a week before the experiment. During this period, they were given cage feeding, sufficient food and water and natural photoperiodic environment to eliminate the influence of artificial photoperiodic factors on visual function. The experimental cats underwent strict eye screening to eliminate the impact of eye diseases on the experimental results. Ketamine hydrochloride was injected at the weight of 40 mg / kg until the animals were deeply anesthetized. Cardiac perfusion was immediately performed on the animals (0.9% saline water). When pale liver was observed, inject 0.1 mol/l PBS (Phosphate Buffered Saline) containing 10% formalin and 2.5% glutaraldehyde (200 ml/kg body weight, pH7.2–7.4) to pre fix the tissue. The brain stem was removed by craniotomy and fixed in the above pre fixation solution for 2 h. The primary visual cortex was cut coronally, and the tissue block was transferred into 30% sucrose solution (containing 2.5% glutaraldehyde and 10% formalin) till the tissue sank to the bottom. Every five continuous coronal frozen slices (50 μm) were separated from each other by an interval of three slices as a set. Every cat included 10 sets of slices. 80 sets of slices were numbered and marked respectively, according to the individuals in the young group and the old group. The five-slice sets were used for Nissl staining, BDNF and TrkB immunohistochemistry labels and negative controls.

The general equipment and reagents used in the experiment are from the food and bioengineering Laboratory of Wuhu Institute of Technology.

### Neuronal Nissl staining

Slices were stained in 0.1% cresyl violet solution for 5 min at room temperature, rinsed with distilled water, dehydrated by gradient alcohol, transparentized by xyl-ene and then sealed with gum. Stained slices were used to determine the layering structure of the primary visual cortex and to provide reference for immunostaining observation and counting.

### Immunohistochemical labeling

The frozen sections were incubated in 3% H_2_O_2_ and rinsed with distilled water, incubated in 0.3% TritonX-100 PBS solution, incubated in 5% fetal bovine serum protein, incubated in Rabbit-anti-mouse BDNF (Catalog Number: PB9075) and Rabbit-anti-human TrkB (Catalog Number: PB0475, GP145-TrkB, full-length antibody with signal transduction activity) primary antibody (1:100, primary antibody) at 4 °C, incubated in secondary antibody (goat-anti-rabbit IgG working solution) at room temperature, incubated in SABC(strept avidin–biotin complex) reagents (third antibody, which were added dropwise) at room temperature, developed by DAB(Diaminobenzidine) to produce colorimetric end products, dehydrated by gradient (80%, 95% and 100%) alcohol, transparentized by xylene and then sealed with neutral gum. The only difference in the treatment of the negative control slices was replacing primary antibodies with PBS. All immunohistochemistry kits and the DAB substrate were products of Boster, Wuhan, China.

### Statistical analysis

40 sets of slices were selected from young cats (20 sets) and old cats (20 sets) (4 cats in each group, and 5 sets of slices were randomly selected for each cat). Nissl-stained, BDNF and TrkB immune-reactive slices were selected and observed under Olympus bx-51 microscope from the young and old groups. The images were collected by Image-Pro express 6.0 software. All the relevant morphological parameters were quantitatively analyzed. The density of BDNF and TrkB immune-reactive cells (evaluated by the number of positive cells per unit area, n/mm^2^) and the intensity of immune-reactions (evaluated by average absorbance value). The average absorbance value is equal to the sum integral optical density divided by the unit area, where higher absorbance values indicated stronger immuno-reactions.

Nissl-stained, BDNF and TrkB immuno-reactive slices were placed at low ocular magnification (40 ×) to obtain the effective image. And then adjust the microscope to ocular magnification (100 ×) to acquire the images of the layering structures. And finally adjust the microscope to high ocular magnification (400 ×), select 10 visual fields (50 μm × 50 μm) of each layer (refer to the layering structure shown in Nissl-stained slices in the same group) to count the number of BDNF and TrkB immunopositive cells and calculate their density (cells/mm^2^). The standard for labeling a BDNF or TrkB immunopositive cell is that the cell body contains obvious immuno-positive matter and a clear nucleus in soma. After collecting images from 20 randomly selected visual fields of each immune reaction slice, the average absorbance value is measured with the help of Image-Pro express 6.0 software, and the mean value is taken as the index indicating the intensity of immuno-reactions. The observer and counter were unaware of the experimental grouping and treatment to ensure data analyses were performed unbiased.

The collected data were analyzed via SPSS 13.0 software and expressed as means ± *SD*, *One-way ANOVA* with *P* < 0.01 being considered statistically significant, the least significant difference (LSD) method was used for multiple comparisons among means.

### ARRIVE guidelines

The study was carried out in compliance with the ARRIVE guidelines

## Results

### Nissl-stained neurons

**Figure 1:**
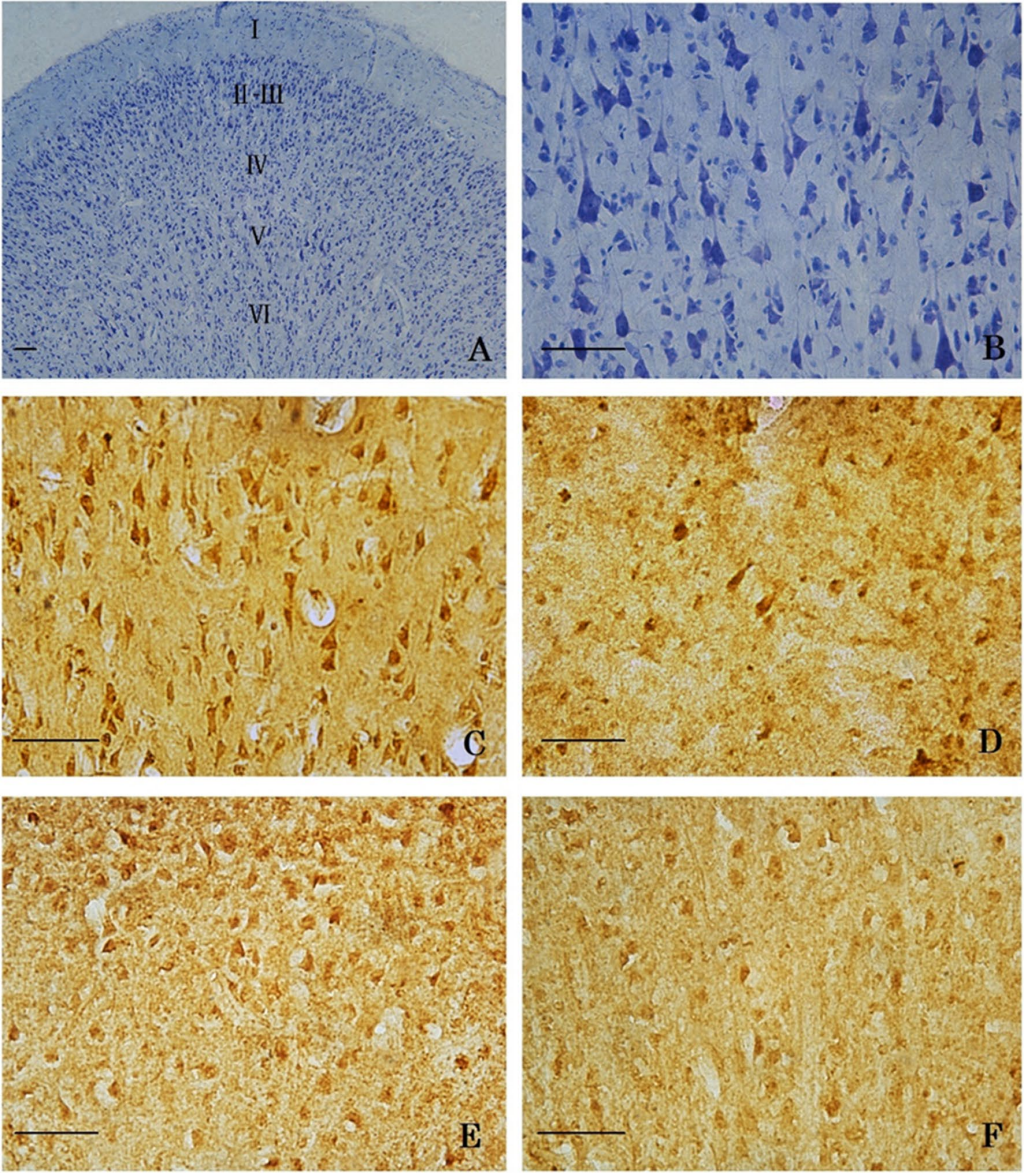
A and B show Nissl-stained layering structure (**A**) (the primary visual cortex can be divided into six layers inward from the surface : molecular layer I, outer granular layer II, outer pyramidal layer III, inner granular layer VI, inner pyramidal layer V and pleomorphic layer VI) and pyramidal neurons (**B**: layer III) in the primary visual cortex. C, D, E, and F show BDNF-IR cells (**C**, **D**) and TrkB-IR cells (**E**, **F**) in the primary visual cortex (layer II-III) of the young cats (**C**, **E**) and the old cats (**D**, **F**). Scale bar = 50 μm.

A six-layer-structure of the primary visual cortex (which can be divided into six layers inward from the surface of the primary visual cortex: molecular layer I, outer granular layer II, outer pyramidal layer III, inner granular layer VI, inner pyramidal layer V and pleomorphic layer VI, respectively) was shown in the Nissl-stained slices. The hierarchical structure of the primary visual cortex and Nissl-body are clear, the morphology and size of neurons are different, and the cell bodies and processes were dyed light blue, blue or blue purple in each layer (such as pyramidal neurons in layer III) (Fig. 1: A, B).

### Densities of BDNF and TrkB immune-positive cells

The brown or dark brown somas of BDNF and TrkB immuno-positive cells and their fiber distributions can be seen in each layer of the primary visual cortex of young and old cats (Fig. 1: C, D, E, F).

Age-independently, the densities of BDNF immuno-positive cells in layer II-III, V and VI were both significantly higher than that of layer I and IV (*F*_(4,195)_ = 32.02, *P* < 0.01), whereas, those between layer I and IV were significantly different(*D-value* > *LSD*_0.01_, *P* < 0.01), and those among layer II-III, V and VI were comparable (*D-value* < *LSD*_0.05_, *P* > 0.05). However, although no differences were found in the densities of BDNF immuno-positive cells in each layer among cats at similar ages (*F*_(3,196)_ = 1.92, *P* = 0.13), those in old cats were significantly lower than those in young cats (*F*_(1,198)_ = 75.43, *P* < 0.01) (Table 1).

**Table 1:** A comparison on density of BDNF and TrkB immuno-positive cells in the primary visual cortex of young and old cats (n = 5, mean ± SD).

subject	Cortical layer				
	I	II-III	IV	V	VI
**The density of BDNF immuno-positive cells (cells/mm**^**2**^**)**					
YC1	382.0 ± 202.6	1723.8 ± 255.3	1422.5 ± 205.4	1852.3 ± 257.5	1868.5 ± 263.5
YC2	362.8 ± 195.3	1658.4 ± 237.4	1388.6 ± 147.5	1774.5 ± 215.3	1714.4 ± 224.3
YC3	397.4 ± 188.4	1888.6 ± 235.2	1448.3 ± 189.2	1769.2 ± 263.4	1694.5 ± 198.4
YC4	357.8 ± 204.3	1525.2 ± 190.8	1523.4 ± 203.5	1655.4 ± 231.4	1720.3 ± 206.4
mean	375.0 ± 188.3^c^**	1699.0 ± 263.5^a^**	1445.7 ± 90.2^b^**	1762.9 ± 245.2^a^**	1749.4 ± 283.5^a^**
OC1	362.5 ± 184.2	1428.8 ± 282.3	1188.6 ± 255.4	1520.6 ± 271.4	1523.8 ± 213.5
OC2	320.6 ± 188.5	1323.2 ± 205.7	1322.3 ± 240.5	1393.3 ± 220.6	1433.2 ± 240.8
OC3	325.6 ± 207.2	1525.0 ± 276.8	1228.5 ± 246.3	1448.6 ± 263.5	1555.3 ± 196.3
OC4	330.2 ± 170.8	1422.5 ± 244.5	1256.3 ± 280.9	1552.3 ± 241.5	1600.2 ± 234.5
mean	334.7 ± 193.6^c^**	1424.9 ± 225.5^a^**	1248.9 ± 27.5^b^**	1478.7 ± 230.1^a^**	1528.1 ± 212.5^a^**
**The density of TrkB immuno-positive cells (cells/mm**^**2**^**)**					
YC1	312.4 ± 197.3	1721.8 ± 241.3	1323.5 ± 223.8	1825.6 ± 206.9	1811.6 ± 195.4
YC2	356.4 ± 168.1	1544.9 ± 230.9	1542.5 ± 200.7	1747.4 ± 192.3	1745.8 ± 230.4
YC3	300.6 ± 195.0	1600.5 ± 240.5	1427.6 ± 189.7	1588.3 ± 195.2	1656.3 ± 176.7
YC4	341.2 ± 187.5	1625.3 ± 210.3	1523.4 ± 192.3	1600.2 ± 187.3	1699.3 ± 182.4
mean	327.1 ± 198.5^c^**	1623.2 ± 191.5^a^**	1454.3 ± 190.2^b^**	1690.3 ± 180.3^a^**	1728.3 ± 265.4^a^**
OC1	286.7 ± 206.3	1518.8 ± 262.2	1280.5 ± 201.3	1448.1 ± 280.9	1591.5 ± 219.5
OC2	312.8 ± 193.5	1610.9 ± 258.1	1310.4 ± 214.6	1299.3 ± 240.8	1503.7 ± 219.3
OC3	296.3 ± 187.5	1555.0 ± 271.7	1338.5 ± 198.3	1347.8 ± 216.5	1653.3 ± 189.7
OC4	320.7 ± 168.4	1487.9 ± 245.5	1276.9 ± 272.8	1522.9 ± 256.2	1458.0 ± 201.2
mean	304.2 ± 198.1^c^**	1518.2 ± 195.5^a^**	1248.9 ± 217.9^b^**	1404.5 ± 190.4^a^**	1551.6 ± 201.2^a^**

YC1-4 and OC1-4 represent different individual from young and old cats respectively. I, II-III, IV, V and VI denote respectively in the primary visual cortical layers.

The different lower-case letters indicate the density of BDNF or TrkB immuno-positive cells in this layer was significantly different from that in other layer (*One way ANOVA*, *LSD-way*, *P* < 0.01).

**Indicates the density of the BDNF or TrkB immuno-positive cells was declined significantly in the old cats when compared with the young cats (*One way ANOVA*, *P* < 0.01).

Age-independently, the densities of TrkB immuno-positive cells in layer II-III, V and VI were both significantly higher than that of layer I and IV (*F*_(4,195)_ = 48.13, *P* < 0.01), whereas, those between layer I and IV were significantly different(*D-value* > *LSD*_0.01_, *P* < 0.01), and those among layer II-III, V and VI were comparable (*D-value* < *LSD*_0.05_, *P* > 0.05). However, although no differences were found in the densities of TrkB immuno-positive cells in each layer among cats at similar ages (*F*_(3,196)_ = 1.86, *P* = 0.14), those in old cats were significantly lower than those in young cats (*F*_(1,198)_ = 114.22, *P* < 0.01) (Table 1).

The statistical results between the groups showed that the density of BDNF and TrkB immuno-positive cells in each layer of primary visual cortex of old cats decreased significantly compared with that of young cats. The BDNF immuno-positive cells density of old cats declines averagely by 10.7% in layer I, 16.1% in layer II-III, 13.6% in layer IV, 16.1% in layer V and 12.7% in layer VI respectively. The TrkB immuno-positive cells density in each layer decrease averagely by 7.0% in layer I, 6.5% in layer II—III, 14.1% in layer IV, 16.9% in layer V and 10.2% in layer VI(Table 1).

### Immuno-intensities of BDNF and TrkB immune-positive cells

**Figure 2:**
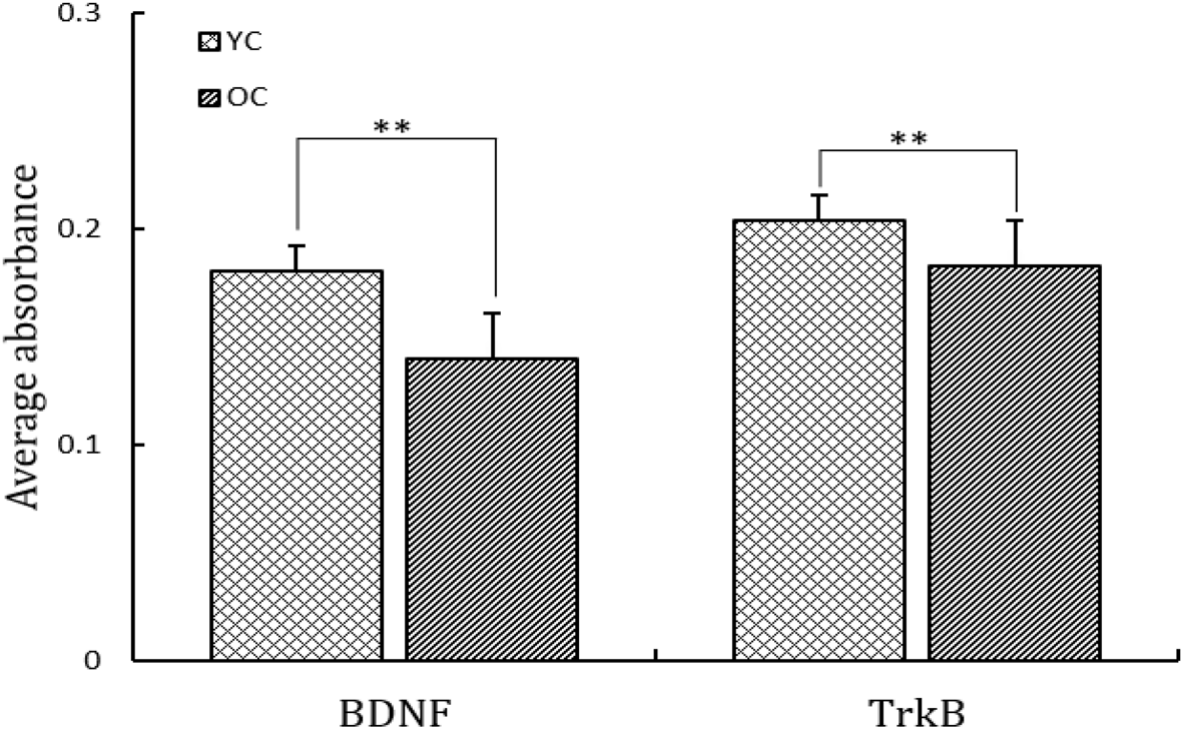
Show the average absorbance value of BDNF and TrkB immunoreactivity in the primary visual cortex of young and old cats. The average absorbance value in old cats was significantly declined when compared with that in young cats (**P < 0.01).

Compared with young cats, the average absorbance value of BDNF and TrkB immunoreactivity in old cats decreased significantly (*F*_(1,38)_ = 58.26, *F*_(1,38)_ = 42.14, *P* < 0.01, Table 2), with a decrease range of 22.2% and 10.3% respectively (Fig. 2).

**Table 2:** A comparison on the average optical absorbance value of BDNF and TrkB immunoreactivity in the primary visual cortex of young and old cats (mean ± SD).

Group	n	BDNF	TrkB
YC	20	0.18 ± 0.06	0.20 ± 0.10
OC	20	0.14 ± 0.14**	0.18 ± 0.07**

**Indicates the average optical absorbance value of BDNF or TrkB immunoreactivity in the primary visual cortex was declined significantly in the old cats when compared with the young cats (*One way ANOVA*, *P* < 0.01).

## Discussion

BDNF is an important member of Nerve Growth Factors and the most widely distributed neurotrophic factor in the brain. It plays an important role in regulating the activities of the nervous system by selectively binding to its specific receptors. Immunohistochemical studies found that BDNF and TrkB, which widely distributed in adult brain tissues, can be secreted by neurons and non-neurons (such as glial cells) in the brain^12^. The visual system is the main nervous system for higher mammals to obtain external information. The three-level structure of retina, lateral geniculate nucleus and visual cortex forms a channel to transmit and process visual information. The functions of visual cortex can decline with aging, typically characterized by the decrease of visual acuity, contrast sensitivity and a selective decline of visual stimuli^13^. The change of BDNF functions can be observed in some neurodegenerative diseases, and there is evidence that the insufficient or decreased density of BDNF is an important cause of neuronal injury, apoptosis and the decline of neuronal synaptic plasticity^14–18^. The protein expression mediated by BDNF-TrkB signal pathway is related to nerve repair and regeneration.

The immunolabeling results of the study systematically reflected the age-related expression differences of BDNF and TrkB in neurons and glial cells in cats’ primary visual cortex. BDNF and TrkB were expressed in all layers of cats’ primary visual cortex, and the expression of BDNF and TrkB in the old group was lower than that in the young group. Indeed, in our experiment, while neurons were specifically immunolabeled, some glial cells were also immunolabeled. It has been reported that glial cells not only support neuronal activity, but also participate in the regulation of neural network plasticity. Astrocytes, microglia and oligodendrocytes play complex and important roles in synaptic plasticity. More and more studies have shown that it is closely related to the occurrence of a variety of neurodegenerative diseases, such as Alzheimer's disease, Parkinson's disease, multiple sclerosis and so on^19–21^.

This result is consistent with the down-regulation of BDNF aging expression in the serum of patients’ with Alzheimer's disease and the aging changes of BDNF and TrkB in various layers of the lateral geniculate nucleus of cats^22,23^. Studies indicate that BDNF plays an important role in nutritional support for the survival and functional expression of GABAergic neurons^24–27^. Previous reports showed that there was no significant change in the total number of neurons in each layer of cats’ primary visual cortex in the elderly group compared with the young group, and the number of GABA positive cells in cats’ primary visual cortex decreased with age^28^. Based on these results, we speculate that the decline of visual function in the visual cortex may not be caused by the decrease of the number of neurons, but more likely by the decline of synaptic plasticity of special neurons mediated by neurotrophic factors. The insufficient inhibitory regulation of GABAergic neurons in primary visual cortex may be the result of the declining synaptic connection mediated by BDNF—TrkB signaling pathway. The possible influence mechanism tends to be like this, the insufficient secretion of BDNF and TrkB in elderly individuals can cause the decline of the morphological and functional stability of inhibitory synaptic connections, which will result in a visual recession characterised by the loss of final visual information transmission.

In summary, the expression of BDNF and TrkB in cats’ primary visual cortex decreased with age, suggesting that the change of BDNF-TrkB signal pathway (such as neuronal apoptosis and synaptic plasticity) caused by the decline of brain-derived neurotrophic factors’ activity may be one of the important causes of visual recession. We look forward to verification in further morphological and electrophysiological experiments.

## Data Availability

All data generated or analysed during this study are included in this published article**.**
